# Carbon footprint assessment and reconstruction redesign of recycled discarded military training uniforms

**DOI:** 10.1038/s41598-025-87733-x

**Published:** 2025-04-01

**Authors:** Ge Huang, Sheng Shi, Qiaoling Wang, Fei Li, Xiaoyan Li, Feng Liu, Zhiwen Lu

**Affiliations:** 1 College of Textile Engineering, Taiyuan University of Technology, Jinzhong, 030600 Shanxi China; 2 Key Laboratory of Waste Polyester Cotton Textiles for Cleaning and Regeneration in Textile Industry, Taiyuan University of Technology, Jinzhong, 030600 Shanxi China; 3 Anhui Tianzhu Textile Science and Technology Group Co.,Ltd, Fuyang, 236000 Anhui China

**Keywords:** Clothing reconstruction and redesign, Carbon rootprint, Discarded military training uniforms, Clothing lifecycle, Recycling, Environmental impact, Engineering

## Abstract

In response to the problem of resource waste and environmental pollution caused by the large amount of waste textiles in China, taking waste military training uniforms as an example, a thorough investigation was conducted to draw a life cycle diagram of military training uniforms, establish a recycling system for waste military training uniforms, and use Taiyuan University of Technology as a pilot for recycling. The carbon footprint of different recycling methods was calculated. A systematic method for the reorganization and redesign of discarded clothing, starting from clothing styling, has been developed. Utilizing discarded military training uniforms and other discarded textiles, practical applications have been completed in seven aspects: silhouette, style, segmentation, clothing components, adding decorations, removing decorations, and Completely redesigned. Finally, based on the problems encountered during the pilot recycling and the insights gained from the restructuring and redesign, a derivative theoretical study was completed. Through the study of the carbon footprint of clothing lifecycle and recycling stages, the importance of reusing discarded clothing has been clarified in the form of data. The innovative “1A2B3C” principles for the recycling and redesign of discarded clothing have been proposed, providing a solid theoretical basis and practical experience for the secondary transformation of discarded clothing. The reuse of discarded military training uniforms has been promoted, and the lifecycle of military training uniforms has been extended from the source of the masses.

## Introduction

Waste textiles refer to the waste textile materials and products in the production and use process. There are roughly three types of waste textiles: first, waste silk, scrap and leftovers in the process of manufacturing textiles; Second, clothing, discarded bedding, curtains, carpets, etc. eliminated after consumption; Third, polyester bottles and other waste plastics with utilizable value^1^. Today’s fast fashion brands have the advantages of large-scale production and low-cost labor. With low-cost fashion products, consumers are encouraged to buy new styles of clothing more frequently, resulting in the shortening of the overall life cycle of clothing and the increase of overall carbon emissions. In 2022, the special document on waste textiles “Implementation Opinions on accelerating the recycling of waste textiles” issued at the national level for the first time has played a role in promoting the development of waste textiles recycling industry^2^. According to the implementation opinions, the recycling rate of waste textiles will reach 25% by 2025 and 30% by 2030. At the same time, it is required to make breakthroughs in the implementation of standard clothing and increase the recycling of waste military uniforms, school uniforms and other uniforms.

With the rapid increase of people’s demand for the upgrading of textiles, the production rate of waste textiles has risen sharply. At present, experts have pointed out many problems in the process of waste textiles from recycling to reuse, and put forward many suggestions such as formulating industry norms and standard systems^3^. In the research on carbon footprint of textile and clothing, some scholars calculated and evaluated the carbon footprint of the production process of raw silk and silk wool products^4^. In the clothing re-engineering, there are not only the research on the selection and use of new materials combined with the re-engineering and re-use design^5^, but also the research on the shape, decoration, color and fabric through the analysis of the re-engineering works of various brands and designers^6^, as well as the exploration on the combination of multi wearing design and clothing re-engineering^7^. Therefore, the recycling and reuse of waste textiles has great research value and development prospects.

It is reported that in 2023, the total enrollment scale of ordinary universities and vocational colleges in China will be 10 million people. Calculated based on the quantity of at least one set per person, the minimum number of military training uniforms that need to be produced and prepared each year is 10 million sets. According to Xinhua News Agency, military training uniforms are usually discarded within a month after their completion. At present, many universities do not have dedicated functional departments responsible for the recycling and disposal of military training uniforms, but rely on student clubs or volunteer organizations for recycling. These organizations have relatively weak recycling systems, authority, and recycling efforts, making it difficult to form effective recycling mechanisms. Meanwhile, due to the relatively simple design and usage scenarios of military training uniforms, many consumers are unwilling to wear uniforms that have been used by others, which limits the reuse rate of military training uniforms. As the main consumer group of military training uniforms, college students regard the recycling and reuse of military training uniforms as an environmentally friendly and resource-saving behavior, and are willing to donate the military training uniforms that are no longer needed to those in need or organizations, in order to contribute their own strength.

Through the research on the life cycle of military training clothing and the evaluation of its carbon footprint after waste recycling, this paper clarifies the importance of waste clothing recycling, puts forward the systematic method of waste clothing re-engineering and carries out the application practice, and finally completes the derivative theory research, which provides a certain reference for the recycling and re-engineering of waste clothing, in order to improve the use value of textiles in the fast fashion dominated modern society, and achieve the ultimate goal of prolonging its use time, life cycle and reducing carbon emissions.

## Materials and methods

The paper adopts a comprehensive research method. Firstly, through lifecycle analysis, a systematic study was conducted on the entire process of military training from design to recovery; Subsequently, data on the usage and recycling needs of military training uniforms were collected through methods such as questionnaire surveys, on-site investigations, and online interviews; Based on these data, a recycling system for used military training uniforms was established, and the carbon footprint of different recycling methods for used military training uniforms was calculated using formulas based on previous research on the carbon footprint assessment of waste textile recycling; Then, a systematic method for the reorganization and redesign of discarded clothing was summarized, and the application practice of reorganization and redesign was carried out using the discarded military training uniforms from Ethereum University of Technology as an example; Finally, based on practical experience and the problems encountered during the recycling process, the “1a2b3c” principles for the recycling and redesign of waste clothing were proposed, providing a solid theoretical basis for the recycling of waste textiles.

### Military training uniform

The military training of Chinese college students is carried out in accordance with the military service law of the people’s Republic of China and the decision of the CPC Central Committee on the reform of the educational system. During the military training, students are required to wear uniform camouflage uniforms, that is, military training uniforms. However, after military training, military training uniforms are rarely worn again. The reuse method is relatively simple, and most of them are discarded and wasted. Therefore, how to recycle military training uniforms after military training has become a major problem^8^.

## Life cycle of military training uniform

**Fig. 1:**
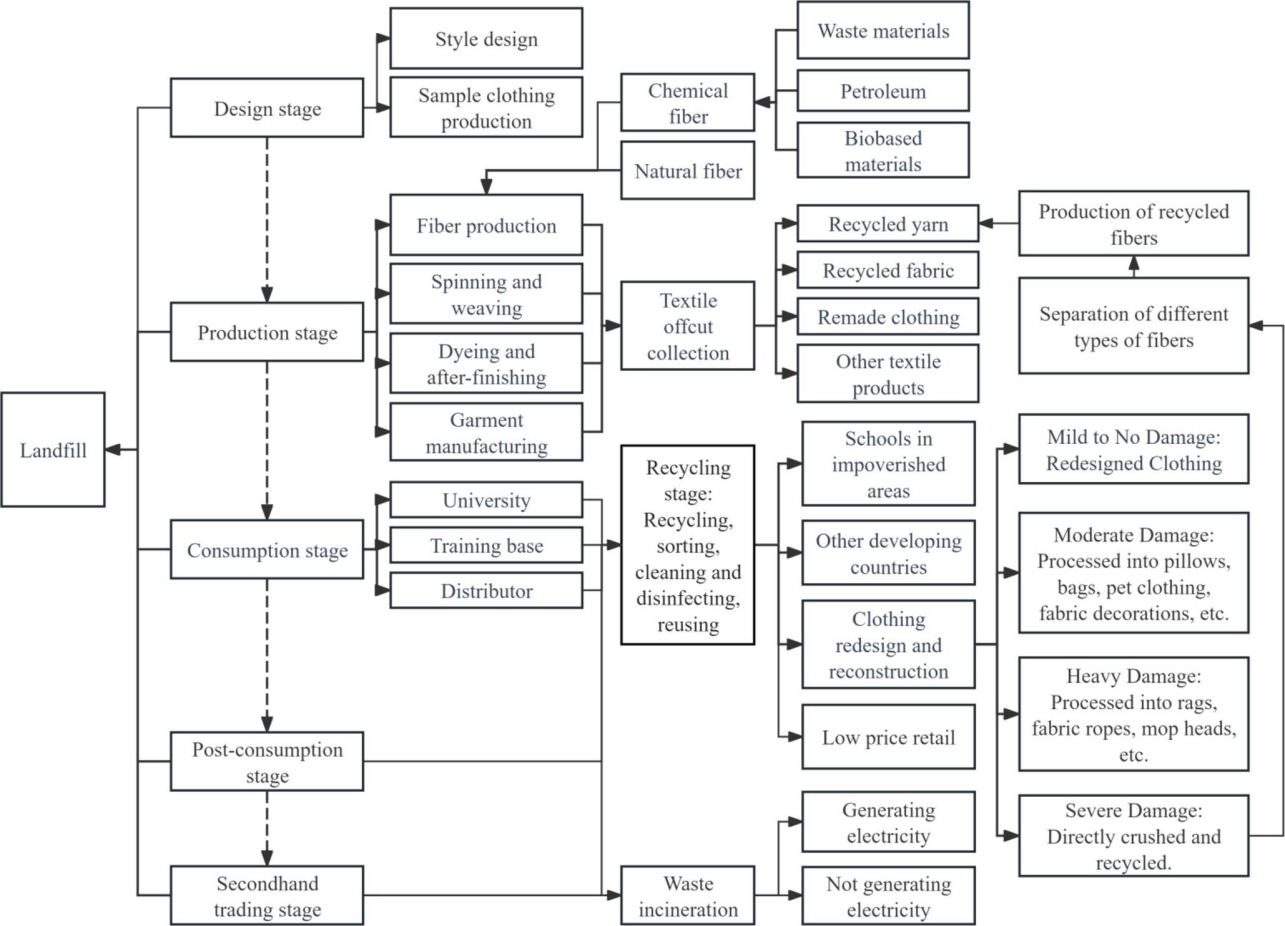
Schematic Diagram of the Lifecycle of Military Training Uniforms.

A product starts from raw material mining, goes through raw material processing, product manufacturing, product packaging, transportation and sales, then is used, recycled and repaired by consumers, and finally recycled or treated and disposed as waste. The whole process is called the product life cycle^9^. This paper studies the life cycle of military training clothing through questionnaire survey, field exploration, online interviews and other methods, and draws a schematic diagram of the life cycle of military training clothing, as shown in Fig. 1.

The life cycle of military training clothes is divided into six stages, starting from the design and development, after production, consumption, post consumption and second-hand trading, the final recycling is carried out, accompanied by waste incineration and landfill. In its whole life cycle, the most visible main environmental impact is solid waste. Only in terms of the recycling stage of waste military training clothing, it can effectively solve the most fatal hard injury of military training clothing, that is, “solid waste” problem.

According to the Research Report^10^ published by the University of Cambridge, UK, the energy consumption of a 100% cotton T-shirt in the whole life cycle is about 109 MJ, and the energy consumption of a 100% viscose fiber blouse in the whole life cycle is about 51 MJ. According to the analysis of the report, in the life cycle of clothing, the energy consumption in the raw material stage, production stage and use stage is more, while the energy consumption after waste is negative, which greatly reduces the waste of resources, so the importance of recycling of waste clothing is self-evident.

## Material composition of military training uniform

**Fig. 2:**
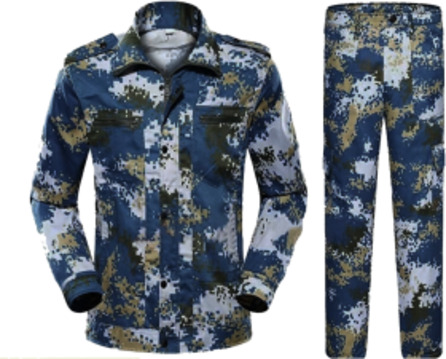
Military Training Uniforms at Taiyuan University of Technology.

Taking Taiyuan University of technology as an example, students’ purchases and distribution of military training supplies include T-shirts, coats, trousers, belts, hats, etc. the colors and patterns are mainly blue camouflage. The style is loose, the design is relatively simple and practical. The fabric of T-shirt, coat and trousers is 100% polyester fiber, which is easy to wash, washable, fast drying, wear-resistant and wrinkle resistant, so as to adapt to high-intensity military training activities. In the recycling of waste military training clothes of Taiyuan University of technology in the following article, considering the daily wearing of T-shirts and the complexity of the components of belts and hats, the recycling focus is on coats and trousers, and the recycled coat and trousers are called a set of military training clothes, as shown in Fig. 2.

## Recycling and carbon footprint assessment of waste military training clothes

It is understood that in 2023, the total enrollment scale of general and vocational colleges across the country was 10 million. Based on the purchase price of each set of military training clothes exceeding 100 yuan, at least billions of yuan of waste military training clothes are faced with the problem of recycling every year, and only a few schools or organizations can uniformly organize and solve the problem of waste military training clothes. In this study, the waste military training clothes of ether University of technology were taken as an example to complete the recycling of waste military training clothes by establishing a recycling system, and calculate the carbon footprint data of different recycling methods.

## Carbon footprint analysis of clothing recycling stage

Carbon footprint (global warming potential) refers to the total amount of greenhouse gases (mainly carbon dioxide) produced by individuals, organizations, products or services in their life cycle, expressed in CO_2_ equivalent ( CO_2_-eq )^11^. Carbon footprint assessment is a part of sustainable development. Through the assessment, we can have a more comprehensive understanding of its environmental impact in different life cycle stages, help to identify key sources of carbon emissions, and then formulate targeted emission reduction strategies.

When carrying out carbon footprint assessment and analysis, it is first necessary to clarify the calculated carbon footprint boundary. In industrial products, according to the provisions of ISO 14,067, carbon footprint boundaries can be divided in many ways, such as “cradle to gate”, “cradle to grave”, “gate to gate”, and some custom carbon footprint boundaries^12^. Different from the mining and production stage of raw materials, energy and auxiliary materials and the processing and production stage of products, when textiles are in the waste treatment stage, CO_2_-eq can be reduced, which corresponds to the negative energy consumption in the waste stage of the whole life cycle of clothing. At the same time, the research shows that CO_2_-eq varies with different recycling methods^13^, as shown in Table 1.The types of waste disposal are divided into four categories: reuse, recycling, incineration, and landfill, each corresponding to different disposal methods: reused waste includes three disposal methods, with no/minor damage being used as redesigned clothing, moderate damage being processed into pillows, bags, pet clothing, fabric decorations, etc., and severe damage being processed into rags, ropes, mops, etc.; The recycled waste is used for the production of regenerated fibers; burn Class of waste is divided into two types: non electricity generating and electricity generating; The waste disposed of in landfills is the same as that of regular garbage.

**Table 1:** Carbon footprint analysis of waste clothing recycling.

Waste treatment type	Reuse			Recycling	Incineration		Landfill
Disposal methods	None/slight damage	moderate damage	severe damage	Production of recycled fibers	No power generation	Power generation	/
CO_2_-eq (kg) /Textiles per kilogram	−3.42285	(−3.42285, 0)	−2.19603 (Rag)	/	/	/	/
CO_2_-eq (kg) /Cotton fiber per kilogram	/	/	/	0.354722	/	−0.81531	/
CO_2_-eq (kg) /Chemical fiber per kilogram	/	/	/	/	2.032646	0.782504	0.470887

### Establish the recycling system of waste military training clothes

In the pre recovery stage, research and investigation should be carried out to understand the demand, potential market and feasibility of the recycling of waste military training clothes. Through understanding the material, style, manufacturing process and possible pollution problems of military training clothes, the corresponding recycling and treatment scheme can be formulated. Secondly, find partners, establish partnership with other relevant organizations with waste military training clothes, and jointly promote the recycling of waste military training clothes through cooperation and interconnection. At the same time, carry out publicity activities to increase people’s awareness of the recycling of waste military training clothes. Disseminate recycling information to students, teachers, parents and other target groups through social media, school activities, publicity materials and other means, and establish the recycling awareness of target groups. Finally, a recycling point is set up. At this stage, the recycling modes commonly adopted for waste textiles include government supported recycling bins, public welfare organizations, “Internet+” recycling, and independent recycling by brand enterprises. In the school, special recycling bins and collection points can be set up to ensure the visibility and convenience of recycling points and encourage students and staff to actively participate.

In the post recovery stage, appropriate treatment methods are selected according to the material, damage and pollution degree of waste military training clothes. Including second-hand clothing reuse, physical or chemical treatment, energy recovery^14^. Establish contact with relevant partners to ensure that waste military training uniforms can be properly treated and reused. Finally, monitoring and evaluation are carried out to measure the recovery effect and environmental impact, and the recovery system is continuously improved and adjusted according to the results.

## Redesign of military training uniform

This section is based on the systematic reorganization and redesign method summarized from the clothing shape, and the application practice of the reorganization and redesign of waste military training clothes in Taiyuan University of technology. Through specific practice, we get the relevant enlightenment of the re-engineering of waste clothing, and put forward the derivative theory of “1a2b3c” based on the problems in the pilot recycling.

## Systematic redesign method of waste clothing

Modeling is to use specific material to shape a visual plane or three-dimensional object image according to aesthetic requirements. Clothing modeling belongs to the category of three-dimensional modeling^15^, which is also divided into structural modeling and decorative modeling. The research aims to give higher added value to waste clothing through creative and inspired redesign^16^, so as to extend the life cycle of clothing and reduce carbon emissions. Taking the clothing modeling as the breakthrough point, this paper systematically summarizes and summarizes the re design methods of waste clothing. As shown in Table 2, there are three main directions in the reorganization and redesign, namely, the structural direction of clothing modeling, the decorative direction and other textile directions. In the direction of clothing structure, the four categories of silhouette, style, segmentation and components are redesigned; In the direction of clothing decoration, the two categories of adding decoration and subtracting decoration are redesigned; In the direction of other textiles, the clothing is completely split and redesigned.

**Table 2:** Systematic Method for Recombinant Redesign of Military Training Uniforms.

Redesign direction		Redesign method	Redesign scheme
Clothing Styling	Clothing Structure	Silhouettes	A-type, H-type, O-type, X-type, T-type, etc.
		Style	Skirt, Shorts, Vest, Crop Top, Overcoat, Dress, etc.
		Segmentation	Horizontal, Vertical, Diagonal, Arc-shaped, Disordered, etc.
		Clothing Components	Pocket, Sleeve, Hem, Front Closure, etc.
	Clothing Decoration	Add Decorations	Beading, Embroidery, Patchwork, Stitching, Padding, etc.
		Remove Decorations	Hollowing, Edge Styling, etc.
Other Textile Products		Completely redesigned	Pillow, Curtain, Bag, Hat, Tablecloth, etc.

## Results

### Carbon footprint calculation of waste military training clothing recycling

By establishing the recycling system of waste military training clothes, ether University of technology as a pilot carried out the recycling of waste military training clothes, and a total of 732 sets of waste military training clothes (about 200 kg) were obtained from Taiyuan University of technology. According to the classification of the recycled military training uniforms with different degrees of damage in the previous life cycle study, there are about 38% of the non/slightly damaged military training uniforms, 46% of the moderately damaged military training uniforms and 16% of the severely damaged military training uniforms. CO_2_-eq obtained from different waste treatment types can be calculated according to formula (1).

1 $$C =\sum_{i=1}^{4}A_{i}c_{i}M$$

Where, C is the calculation result of CO_2_-eq during the recovery and treatment of waste military training uniforms, in kg; i is the i-th waste treatment method; c_i_ is the CO_2_-eq of the corresponding unit mass (1 kg) of textiles or fibers using the i-th waste treatment method, and the unit is kg/kg (two kg units have different meanings, the former refers to the carbon dioxide equivalent of greenhouse gas emissions, and the latter refers to the textiles or fibers per unit mass); A_i_ is the percentage of the mass of the corresponding textile or fiber in the total mass using the i-th waste treatment method; M is the total mass of textile or fiber, in kg. Through formula (1), the calculation results of CO_2_-eq emitted by different waste treatment types are obtained by combining the data in Table 1 with the actual recovery data, as shown in Table 3.

**Table 3:** Carbon footprint calculation of waste clothing recycling.

Waste disposal type	Waste disposal method	Percentage of corresponding textile or fiber mass in total mass (A_i_)	CO_2_-eq corresponding to textile or fiber emissions per unit mass (c_i_)	Total mass of textile or fiber (M)	Total CO_2_-eq(C)
Reuse	No/slight damage	38%	−3.42285	200	−489.38066
	Moderate damaged	46%	−1.711425 (Take median)		
	Severe damage	16%	−2.19603 (Rag)		
Burn	No power generation	chemical fiber: 100%	chemical fiber: 2.032646	200	406.5292
	Power generation	chemical fiber: 100%	chemical fiber: 0.782504		156.508
Landfill	/	chemical fiber: 100%	chemical fiber: 0.470887	200	94.1174

It can be seen from the data in Table 3 that only when the 200 kg old military training clothes are recycled and reused can the purpose of reducing carbon emissions be achieved, and the CO_2_-eq emissions of about 489.38066 kg can be reduced; In the recycling process, according to the analysis of the actual recycled waste military training clothing fabric composition in the previous article, it is found that it is not suitable for the production of recycled fiber, so it is not calculated; Both incineration and landfill will increase the carbon footprint, especially when incineration does not generate electricity, which will increase CO_2_-eq emissions by 406.5292 kg. If the number of undergraduate freshmen in the University of Ethereum and technology in 2023 is 8253, and one set of waste military training clothes is recycled from each person (based on the actual recycled 732 sets of clothes of about 200 kg, the average weight of each set is about 0.273 kg), and all the waste clothes are reused, the CO_2_-eq emission will be reduced by about 5513 kg. When applied to colleges and universities across the country, the carbon reduction effect is obvious. Therefore, it is of far-reaching significance to redesign the waste military training clothes so that they can be worn and reused daily.

### Application practice of re design of waste military training clothing

From the perspective of clothing structure, in the re design of silhouette, it is mainly divided into type A, type H, type O, type X and type T. through the structural design of splitting, restructuring, splicing and detail adjustment, the clothing silhouette has undergone a variety of changes, so that the old clothing can radiate the charm of fashion with the new silhouette; In the re design of the style, the key is to give full play to creativity and imagination, transform old clothes into distinctive new styles, make further use of old clothes, and bring new possibilities for fashion modeling while reducing waste; In the reorganization and redesign of segmentation, it is divided into structural segmentation lines and decorative segmentation lines, which have various forms such as horizontal, vertical, oblique, arc, and disorder. Through ingenious use of design techniques and fabric replacement, a unique segmentation line effect is created, bringing new visual characteristics and fashion style to the reconstructed clothing; In the re design of parts, new design effects are created through the changes of various parts of clothing, such as changing the collar and sleeve, changing the position, shape and size of pockets, adjusting the shape of hem or placket, etc.

From the perspective of clothing decoration, in the process of adding decoration and re designing, the gorgeous feeling and details of clothing are added by inlaying beads; Inject exquisite and personalized elements into clothing through embroidery; Create interesting and unique visual effects through patchwork; To decorate clothing by stitch method; Through the filling method to create a convex effect, increase the clothing texture and sense of hierarchy. At the same time, appropriate decorative elements should be considered according to the style and fabric of clothing to maintain the balance and coordination of decoration. In the redesign of subtracting decoration, the hollowed out effects of different shapes and sizes are created by means of laser cutting or weaving, and the hollowed out design is applied to different positions, such as neckline, cuffs, back or skirt hem, to add personality and uniqueness; Or through cutting, sewing, bandage, selvage or taping and other technologies to modify the edge of clothing, create a unique clothing contour and shape.

The complete reorganization and redesign of clothing refers to the transformation of old clothing into textiles with different functions, so as to extend the service life and increase the use value, including but not limited to throw pillows, curtains, bags, hats, etc., mainly for waste clothing with moderate damage.

**Fig. 3:**
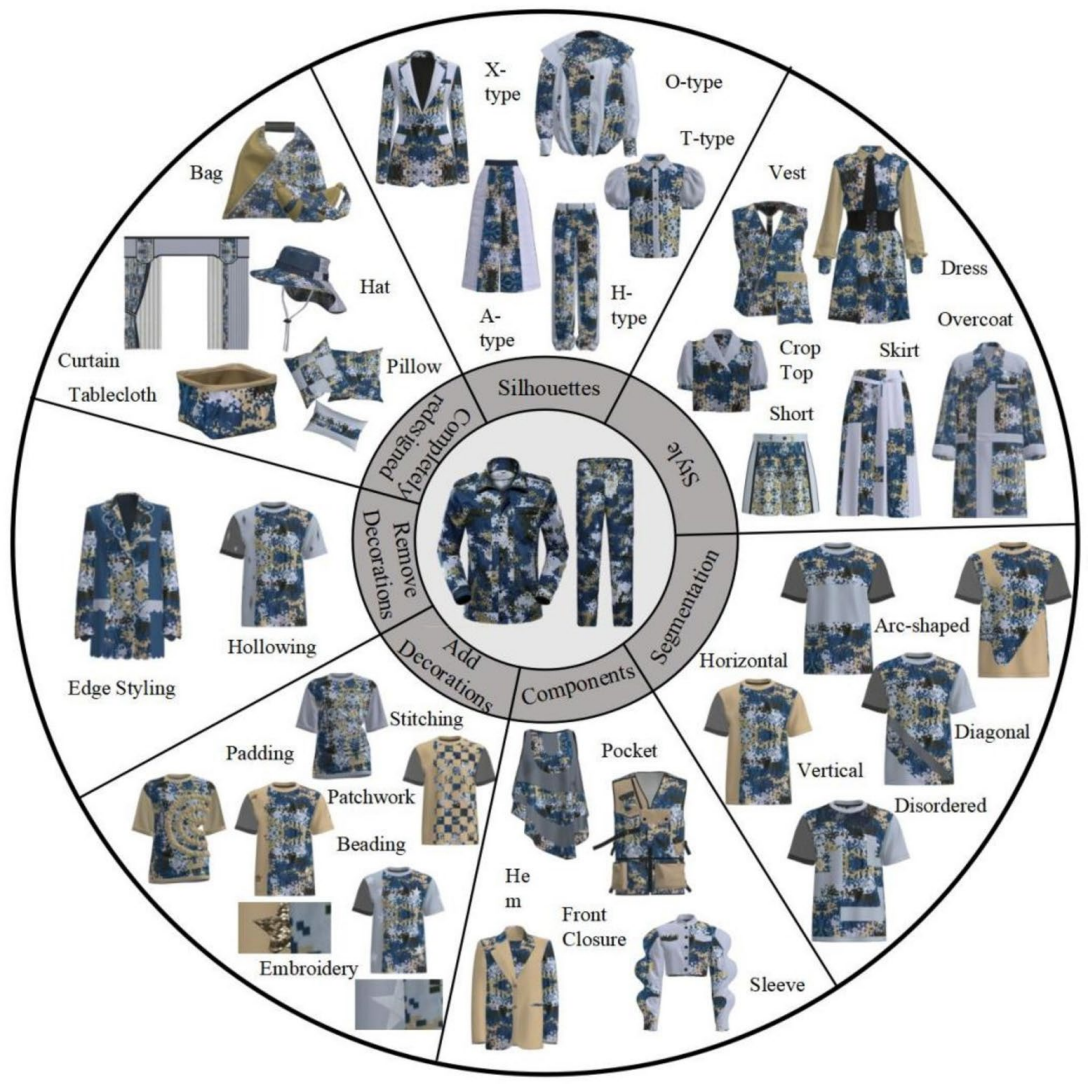
Application of reengineering and redesign.

This section will use the summarized systematic reorganization and redesign method of waste clothing to carry out the secondary design of waste military training clothing. Taking the military training clothing of Taiyuan University of technology as an example, through the application practice with other types of waste textiles, the application effect is shown in Fig. 3 .

### Derived recycling and redesign principle of waste clothing - “1a2b3c”

Combined with the experience of the application of re-engineering in 3.2 and the problems in the pilot recycling of waste military training clothes, six basic principles of “1a2b3c” including aesthetics, backflow, baseline, clean, converting and classification are proposed, in order to provide experience for the recycling and re-engineering of other waste clothing.

In the recycling stage of waste clothing, attention should be paid to the principle of cleanliness and classification. The basic principle of the second use of waste clothing is hygiene and safety. From the perspective of enterprises, charities or other non-profit organizations, waste clothing comes from different groups, covers a wide range and has a large number, so it needs special attention in terms of health and safety. Through batch cleaning, disinfection, sterilization and other methods, to ensure that the odor is removed, and bacteria, viruses, mites and other microorganisms are completely removed, so as to prevent infection or allergy in the case of respiratory tract and skin contact^17^. The classification makes the recycling process more efficient and accurate, and the clothing of different types and materials are treated separately, such as physical disassembly, chemical treatment, etc. Correct classification ensures that each type of clothing is properly treated, improves the efficiency of resource recovery, reduces resource waste, and reduces environmental load. At the same time, the classification of waste clothing can provide more data and information to study and analyze the consumption and market demand of different types of clothing, help guide the sustainable development of the fashion industry, and promote the design and production of more environmentally friendly, durable and consumer friendly clothing.

In the redesign stage of waste clothing, we should pay attention to the aesthetic principle, the return principle, the bottom line principle and the classification principle. Loving beauty and pursuing beauty are the current trend and fashion. Under such a strong demand for beauty, people are more inclined to choose works that can stimulate visual perception and resonate^18^ to promote consumers’ desire to buy and promote the reuse of second-hand clothing, whether it is popular fashion or redesigned clothing. The return flow principle refers to the return flow in the product life cycle. A garment has to go through different life cycle processes from design to acceptance by consumers, and then to circulation and sales. The life cycle of clothing is an overall one-way cycle. When transforming and redesigning clothing, the goal is to increase the return flow in the one-way cycle of clothing life cycle. The length and number of return flow stages are not limited, and one or several stages are repeated to achieve the ultimate goal of prolonging the product life cycle and improving the use value of products. At the same time, in the whole process of redesign and transformation of waste clothing, we should take environment and resources as the ultimate bottom line, and resolutely resist excessive waste of resources and unnecessary environmental pollution, such as unnecessary dyeing and printing of redesigned clothing (waste gas, waste water, waste residue, etc.), serious insufficient fabric utilization (causing a large number of solid waste), etc. The conversion principle refers to the conversion of product categories. Textile products mainly include clothing textiles, home textiles and industrial textiles. In the process of clothing redesign and transformation, we should not stick to the clothing itself, and should take a long-term view. The transformation is equivalent to “pulling” a product out of the life cycle of clothing and “putting” it into the life cycle of another type of product. In essence, it is another way to extend the life cycle.

## Conclusions

This study establishes the core position of second-hand clothing reuse in reducing carbon emissions and promoting resource recycling through in-depth analysis of the lifecycle of military training uniforms, especially the carbon footprint calculation during the waste recycling stage. The study not only summarized systematic methods for the recycling of waste textiles, but also used the waste military training uniforms from Taiyuan University of Technology as a specific case to successfully carry out recycling application practice, verifying the feasibility and effectiveness of these methods. On this basis, we further conducted theoretical innovation and proposed six core principles of “1a2b3c”, providing a comprehensive and operational guidance framework for the recycling and reconstruction of waste clothing, filling the gap in theoretical research in this field.

Although there are certain implementation methods for the recycling and transformation of waste textiles within various organizations, these methods have not been widely promoted and a unified standard has not yet been formed nationwide. However, with the increasing emphasis on the concept of sustainable development in society and the continuous improvement of relevant policies and systems, the recycling and utilization of waste textiles is gradually becoming an important issue in the textile industry. This study firmly believes that with the continuous advancement of technology, sustained policy support, and increasing public environmental awareness, the recycling and utilization of waste textiles will play a more important role in the future textile industry. It can not only effectively reduce resource waste and environmental pollution, but also promote the green transformation and upgrading of the textile industry. Therefore, future research should continue to deepen technological innovation in the recycling and remanufacturing of waste textiles, strengthen policy guidance and standard setting, promote the widespread promotion and application of waste textile recycling, and contribute to the sustainable development of the textile industry.

Looking ahead to the future, there is still vast research space in the field of recycling waste textiles. Firstly, the pilot scope of the waste military training uniform recycling system should be further expanded to verify its applicability in different environments and conditions, and gradually promoted nationwide. Secondly, deepen the research on carbon footprint, refine the environmental impact of various waste textiles in different treatment processes, and provide scientific basis for policy formulation. Meanwhile, technological innovation is the key to promoting the development of this field, and research efforts on new technologies such as biodegradation and chemical recycling should be increased. In addition, understanding and guiding consumer behavior is crucial for improving the recycling rate of waste textiles. Future research should focus on consumer psychology and behavior patterns, and design more effective incentive mechanisms and promotional strategies. Finally, the formulation and improvement of policies and standards are also an indispensable part of promoting the recycling of waste textiles. It is necessary to strengthen interdisciplinary cooperation and jointly promote the healthy development of the industry.

## Data Availability

The data generated during the current study are available from the corresponding author on reasonable request.
